# Islet Transplantation Reverses Podocyte Injury in Diabetic Nephropathy or Induced by High Glucose via Inhibiting RhoA/ROCK/NF-*κ*B Signaling Pathway

**DOI:** 10.1155/2021/9570405

**Published:** 2021-03-10

**Authors:** Chongchu Huang, Yi Zhou, Hongjian Huang, Yushu Zheng, Lijun Kong, Hewei Zhang, Yan Zhang, Hongwei Wang, Mei Yang, Xiaona Xu, Bicheng Chen

**Affiliations:** ^1^Key Laboratory of Diagnosis and Treatment of Severe Hepato-Pancreatic Diseases of Zhejiang Province, The First Affiliated Hospital of Wenzhou Medical University, Wenzhou, 325000 Zhejiang Province, China; ^2^Transplantation Centre, The First Affiliated Hospital of Wenzhou Medical University, 325015 Wenzhou, Zhejiang Province, China; ^3^Intensive Care Unit, The First Affiliated Hospital of Wenzhou Medical University, 325015 Wenzhou, Zhejiang Province, China; ^4^Operating Room, The First Affiliated Hospital of Wenzhou Medical University, 325015 Wenzhou, Zhejiang Province, China

## Abstract

**Objective:**

Abnormal signaling pathways play a crucial role in the mechanisms of podocyte injury in diabetic nephropathy. They also affect the recovery of podocytes after islet transplantation (IT). However, the specific signaling abnormalities that affect the therapeutic effect of IT on podocytes remains unclear. The purpose of this study was to assess whether the RhoA/ROCK/NF-*κ*B signaling pathway is related to podocyte restoration after IT.

**Methods:**

A mouse model of diabetic nephropathy was established *in vivo* using streptozotocin. The mice were then subsequently reared for 4 weeks after islet transplantation to determine the effect of IT. Islet cells, CCG-1423 (RhoA Inhibitor), and fasudil (ROCK inhibitor) were then cocultured with podocytes *in vitro* to assess their protective effects on podocyte injury induced by high glucose (HG). Protein expression levels of RhoA, ROCK1, synaptopodin, IL-6, and MCP-1 in kidney tissues were then measured using immunohistochemistry and Western blotting techniques.

**Results:**

Islet transplantation reduced the expression levels of RhoA/ROCK1 and that of related inflammatory factors such as IL-6 and MCP-1 in the kidney podocytes of diabetic nephropathy. In the same line, islet cells reduced the expression of RhoA, ROCK1, and pp65 in immortalized podocytes under high glucose (35.0 mmol/L glucose) conditions.

**Conclusions:**

Islet transplantation can reverse podocyte injury in diabetes nephropathy by inhibiting the RhoA/ROCK1 signaling pathway. Islet cells have a strong protective effect on podocytes treated with high glucose (35.0 mmol/L glucose). Discovery of signaling pathways affecting podocyte recovery is helpful for individualized efficacy evaluation and targeted therapy of islet transplantation patients.

## 1. Introduction

Diabetic nephropathy (DN) is a serious microvascular complication that causes end-stage renal disease [1]. It is characterized by appearance of progressive proteinuria, glomerular filtration injury, glomerular sclerosis, and glomerular basement membrane (GBM) thickening. Podocytes are essential components of glomerular filtration barrier function. As such, an injury to podocytes results to the development of proteinuria [2]. Podocyte injury is one of the most important mechanisms that lead to DN [3]. Cognizant to this, reversing podocyte injury is a promising therapeutic target for DN patients.

Islet transplantation is a promising alternative approach for type 1 diabetes patients with serious complications [4]. It decreases urinary excretion of albumin, improves microvascular and macrovascular function, and reverses related renal damage [5, 6]. Though blood glucose is controlled at normal levels in all animal models of diabetic nephropathy after islet transplantation, there are individual differences in the reversal of kidney and podocyte injury. It is therefore postulated that abnormal signal activation affects podocyte recovery after islet transplantation [7].

Abnormal signaling pathways play a critical role during the processes thereby leading to podocyte lesions in diabetic nephropathy [8]. The small GTPase that belong to the Ras homology (Rho) family play important roles in regulating a variety of signal transduction pathways such as cell migration, proliferation, and phagocytosis [9]. RhoA is the most common member of the Rho GTPase family. Its major downstream effector is ROCK1. It is also postulated that the RhoA/ROCK pathway contributes to the development of diabetic complications such as diabetic nephropathy [10]. As such, inhibition of this pathway prevents occurrence of pathologic changes of diabetic nephropathy *in vivo* [11].

Herein, both *in vivo* and *in vitro* experiments were conducted to investigate the effects of islet transplantation on podocyte injury. In the same line, the RhoA/ROCK signaling pathway and the NF-*κ*B pathway were analyzed to explore possible protective mechanism of functional islets on podocyte injury. This was done to analyze the causes of poor renal function after islet transplantation which would further aid in development of clinical targeted therapy for islet transplantation.

## 2. Materials and Methods

### 2.1. Animal Models

Mature male C57BL/6 mice (8 weeks old, weighing 18–22 g) were purchased from the Laboratory Animal Center (Wenzhou Medical University, Wenzhou, China) for animal-based models. The mice were housed in specified pathogen free (SPF) environment at 24°C ± 1°C and with a 12-hour light/dark cycle for at least one week prior to the experiment. Twenty mice were then induced to develop diabetes (blood glucose >16.67 mmol/L) through intraperitoneal injection with streptozotocin (STZ) (60 mg/kg, Sigma Aldrich, St. Louis, USA) in sodium citrate buffer for 5 consecutive days. Another seven mice were used as the negative control group. Urinary protein and creatinine of the mice were then assessed using an automatic biochemical analyzer after a stable diabetic status of 18 weeks.

The DN mice were then further divided into two groups: DN group (*n* = 7, mice with diabetic nephropathy) and IT group (*n* = 7, DN mice treated by islet transplantation). A control group (*n* = 7, mice without any treatment) was also established. These experiments were reviewed and approved by the animal policy and welfare committee of the Wenzhou University.

### 2.2. Cell Culture Conditions

Conditionally immortalized podocyte cell lines from mice (National Infrastructure of Cell Line Resources, Beijing, China) were routinely maintained at 33°C and 5% CO_2_ in DMEM medium (5.6 mmol/L D-glucose) supplemented with 10% FBS and 10 units/mL recombinant *γ*-interferon (IFN-*γ*, Sigma). Prior to the experiment, the cells were cultured without INF-*γ* at 37°C for 14 days to induce differentiation.

The differentiated podocytes were plated in 6-well plates and cultured under 9 different conditions to assess the degree of podocyte injury. The nine sets of conditions were 5.6 mM D-glucose as normal glucose group, D-glucose (5.6 mM) + mannitol (29.4 mM), 20.0 mM D-glucose, 35.0 mM D-glucose, 35.0 mM D-glucose + fasudil (10^−6^ mM), 35.0 mM D-glucose + fasudil (10^−5^ mM), 35.0 mM D-glucose+ islet cells (coculture group), D-glucose (5.6 mM) + fasudil (10^−5^ mM), and 35.0 mM D-glucose + CCG-1423 (1 *μ*M).

### 2.3. Islet Transplantation

Male C57BL/6 mice were selected as pancreatic islet donors. The mice were first subjected to isoflurane anesthesia followed by injection with 5 mL collagenase V (GIBCO, CA, USA) into the pancreatic duct via the common bile duct. This was done after ligation at the ampulla of Vater. The pancreases were subsequently inflated and removed and then transferred into a stationary water bath at 37°C for digestion. The islets were then separated through density gradient centrifugation using Histopaque-1077 and handpicked into black glass petri dishes. The pure islets were then cultured in RPMI-1640 (5.6 mM glucose, Gibco, CA, USA) for at least 6 hours and their viability assessed using FDA-PI (Fluorescein diacetate-propidium iodide, Solarbio, Beijing, China) prior to transplantation. Islets of 250–350 islet equivalent (IEQ) were then slowly placed beneath the kidney capsule upon exposure of the kidney [12].

### 2.4. Western Blot Analysis

The Protein BCA Assay kit (Beyotime, Jiangsu, China) was used to determine the total protein concentration of the cells and kidney cortex. The proteins were then separated by size using a 10% SDS-polyacrylamide gel electrophoresis followed by electrophoretic transfer of the resultant bands onto nitrocellulose membranes. The membranes were subsequently blocking with 5% skimmed milk powder supplemented with Tris-buffered saline-Tween buffer and then incubated overnight with primary antibodies at 4°C. The primary antibodies were MCP-1 (Santa Cruz Biotechnology, Inc.), *β*-actin (Cell Signaling Technology, CST, Boston, USA), ROCK1 (CST), GAPDH (CST), RhoA (CST), IL-6 (CST), PP65 (CST), CCG-1423 (Santa Cruz), and synaptopodin (Santa Cruz). The membranes were then incubated with the goat anti-rabbit/mouse immunoglobulin G horseradish peroxidase- (HRP-) conjugated secondary antibody (BioSharp, Technology Inc., China). The resultant banding patterns were analyzed using the Image Lab Software (Amersham imager 680, Cytiva, USA; UVP ChemStudio/PLUS, Analytik Jena AG, Germany).

### 2.5. Immunohistochemistry and Immunofluorescence Staining

Immunoperoxidase procedures were carried out on paraffin-embedded renal tissue sections of 4 *μ*m thickness. The sections were first incubated in hydrogen peroxide (3%) to quench endogenous peroxidase activity after deparaffinization in dimethyl benzene and rehydration in graded alcohols and water. Antigen retrieval in boiling sodium citrate buffer (pH = 6.0) was then done, and the sections subsequently blocked with normal goat serum (5%). The slides were incubated with specific antibodies (synaptopodin, MCP-1, IL-6, ROCK1 and RhoA). Peroxidase-coupled goat anti-rabbit immunoglobulin G (BioSharp Inc., China) served as the secondary antibody and 3,3′-diaminobenzidine (DAB) as the coloring agent. Immunofluorescence staining of mouse kidney tissues was performed using insulin antibody (2D11-H5; Santa Cruz Biotechnology, Dallas, TX) followed by rinsing and addition of FITC-conjugated secondary antibodies (Santa-Cruz). Images were randomly selected for each slide and the micrographs subsequently captured under ×100 and ×200 magnification.

### 2.6. Data Analysis

Data was processed and analyzed using the SPSS 20.0 statistical software (Chicago, IL USA). Data was presented as means ± standard deviation (SD). Differences between groups were determined using one-way analysis of variance (ANOVA). *P* values less than 0.05 (*P* < 0.05) indicated that there were significant differences between groups. All data presented were obtained from independent triple-replicated experiments.

## 3. Results

### 3.1. Evaluation of Islet Transplantation

**Figure 1:**
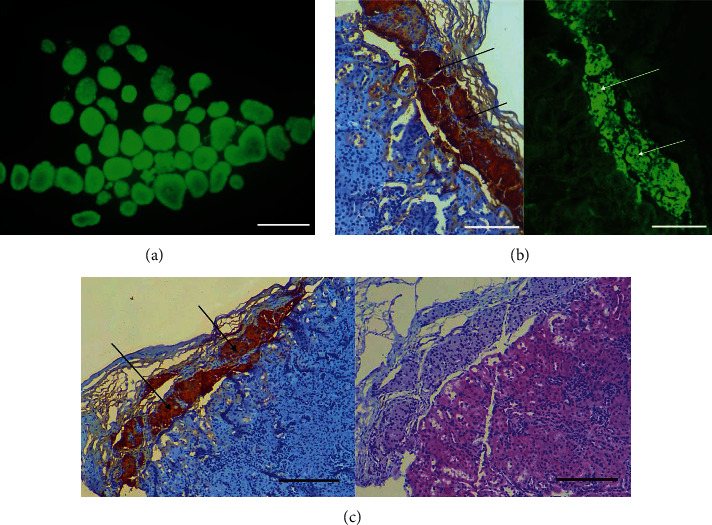
Evaluation of islets vitality, location, and function after transplantation. (a) Viability evaluation of pancreatic islets (FDA/PI staining: ×200). Scale bar = 50 *μ*m. (b) The activity of islets transplanted under renal capsule was high and insulin secretion was normal (The arrow points to the positive expression of insulin antibody, immunohistochemical staining: ×200; immunofluorescence staining: ×200). Scale bar = 50 *μ*m. (c) Transplanted islets were at a high activity under the kidney capsule (The arrow points to the positive expression of insulin antibody, immunohistochemical staining: ×100; HE staining: ×100). Scale bar = 100 *μ*m.

FDA-PI staining was used to evaluate the activity of the islets after extraction of the purified islet cells. The activity of islet cells was high before transplantation (>99%, Figure 1(a)). Hematoxylin-eosin (HE), immunohistochemical, and immunofluorescence staining further revealed that transplanted islets had high activity and still secreted insulin under the kidney capsule (Figures 1(b) and 1(c)).

### 3.2. Construction of Mice Model

**Figure 2:**
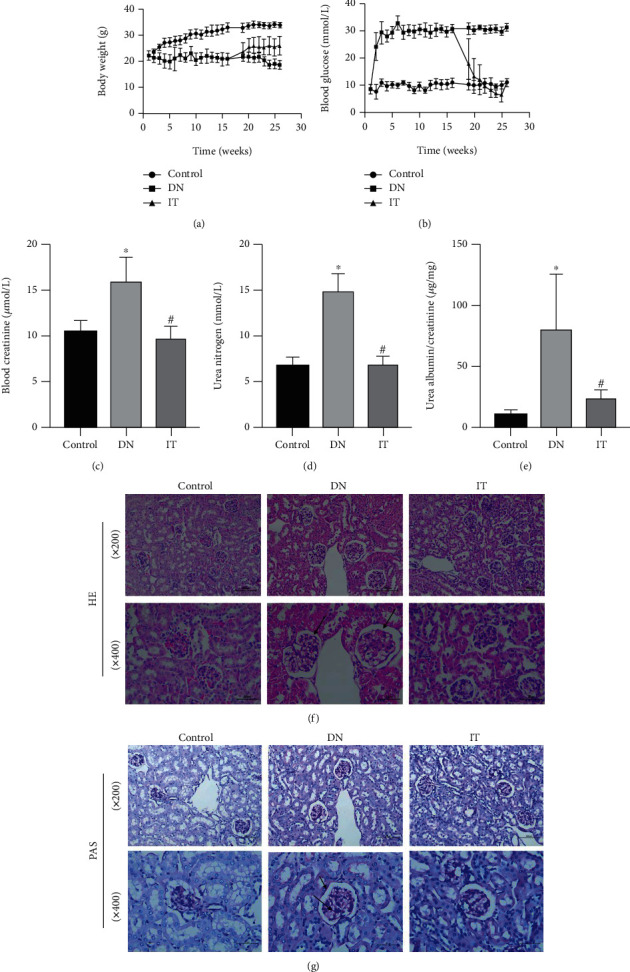
Improvement of body weight, blood glucose, renal function, and pathology in mice after islet transplantation. (a) Random blood glucose was measured once a week (islet transplantation model mice established at 18 weeks). (b) The weight of mice was recorded once a week. (c, d) Blood creatinine and urea nitrogen were measured for each group. (e) Urinary albumin-to-creatinine was measured for each group. (f, g) The HE and PAS staining of glomerular structure showed typical pathological changes (The arrow in HE points to the glomerular hypertrophy; the arrow in PAS points to the glomerular basement membrane thickening) (^∗^*P* < 0.05 versus the control group, ^#^*P* < 0.05 versus the DN group).

Blood glucose level, body weight, urine protein-to-creatinine, urea nitrogen, and blood creatinine were measured to determine whether there were differences between groups. The body weight of mice in the DN group remained constant at low levels while that of mice in the control group rose continuously. Mice in the IT group instantly gained weight after transplantation (Figure 2(a)). In the same line, the blood glucose levels of diabetic mice decreased significantly after transplantation while those of the other two groups remained at the initial levels (Figure 2(b)). In addition, the level of urine protein-to-creatinine, urea nitrogen, and blood creatinine decreased significantly in the IT group compared to untreated DN mice (Figures 2(c)–2(e)). PAS (periodic acid-schiff) and HE staining were subsequently used to analyze glomerular pathological changes in each group. Mice in the DN group had significant renal microstructure injuries (podocyte depletion, glomerular hypertrophy, and glomerular basement membrane thickening) compared with those in the control and IT group (Figures 2(f) and 2(g)).

### 3.3. Islet Transplantation Inhibits Inflammation of Renal Tissues

**Figure 3:**
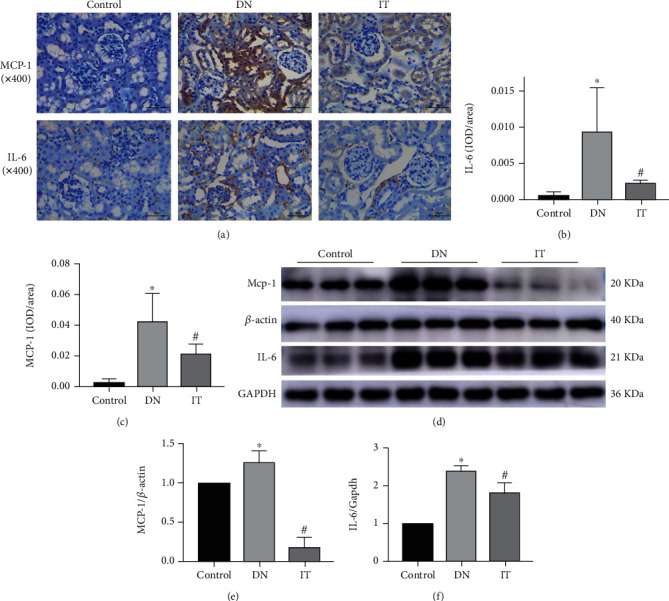
Islet transplantation inhibited the expression of inflammatory factors in diabetic nephropathy mice. (a–c) Immunohistochemical staining (×400) and quantitative analysis showing the expression levels of IL-6 and MCP-1 in different groups. Quantifications of IL-6 and MCP proteins expression in the kidney was measured by mean integrated optical density (IOD)/area. (d–f) Levels of IL-6 and MCP-1 proteins were quantified by western blot analysis. There are three replicates in each group (^∗^*P* < 0.05 versus the control group, ^#^*P* < 0.05 versus the DN group).

IHC (immunohistochemistry staining) and WB (Western blotting) were performed to determine the protein levels of inflammatory-related factors that were further used to assess the effect of islet transplantation on inflammation. MPC-1 and IL-6 were highly expressed in the kidney tissue of the DN group. However, there was no significant expression of the two factors in the control and IT groups (Figure 3(a)). Semiquantitative scores further revealed that the OD values of MPC-1 and IL-6 in the DN group were higher than those of MPC-1 and IL-6 in the control group. Similarly, the OD values of MPC-1 and IL-6 in the IT group were lower than those in the DN group (Figures 3(b) and 3(c)). Western blot analysis also revealed that the expression levels of MPC-1 and IL-6 in the DN group were significantly higher than those in the control group. Moreover, the expression of MPC-1 and IL-6 in the IT group was lower than that of the DN group (Figure 3(d)). WB analysis results were consistent with those of IHC (Figures 3(e) and 3(f)).

### 3.4. Islet Transplantation Reverses Podocyte Injury Caused by Diabetic Nephropathy through Inhibition of the RhoA/ROCK1 Pathway

**Figure 4:**
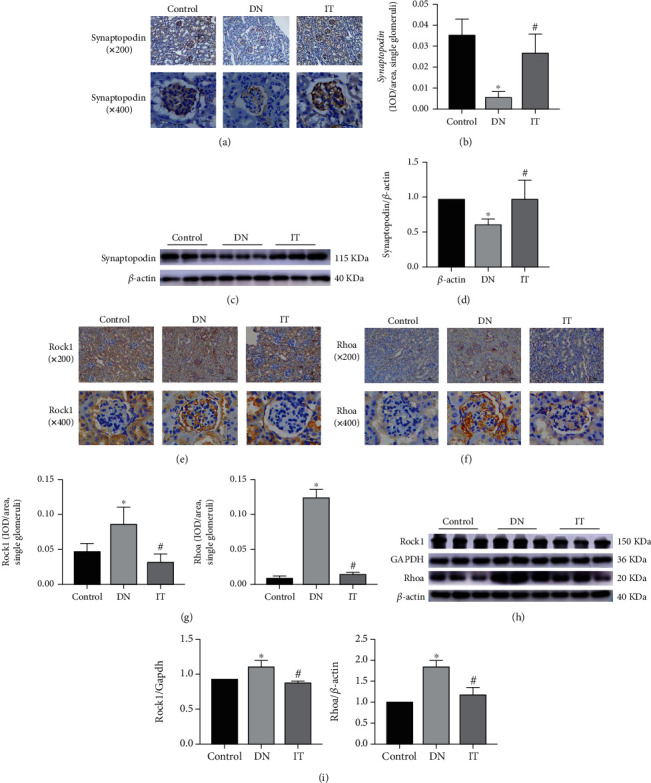
The effects of islet transplantation on synaptopodin and RhoA/ROCK1 proteins in diabetic nephropathy mice. (a, b) Synaptopodin protein was detected by immunohistochemical staining (×200, ×400), and then, podocyte injury and restoration were quantitatively analyzed in different groups. Quantifications of Synaptopodin protein expression in single glomeruli was measured by mean integrated optical density (IOD)/area. (c, d) Western blot was used to detect the expression of synaptopodin protein and quantitatively analyze recovery of podocytes after islet transplantation. (e, f) The expression and distribution of RhoA/ROCK1 proteins in immunohistochemical photographs (×200, ×400) of different kidney tissues. (g) Quantifications of RhoA/ROCK1 proteins in single glomeruli was measured by mean integrated optical density (IOD)/area. (h, i) Western blot and semiquantitative analysis of RhoA/ROCK1 proteins expression in renal tissue (^∗^*P* < 0.05 versus the control group, ^#^*P* < 0.05 versus the DN group).

The recovery of podocytes after islet transplantation was detected by IHC and WB. The proportion of synaptopodin-positive cells was significantly increased in the glomerulus of DN mice after islet transplantation (Figures 4(a) and 4(b)). WB analysis further revealed that there was a significant increase in the podocyte level (synaptopodin) of the IT mice compared to the untreated DN mice (Figures 4(c) and 4(d)). In the same line, the proportion of RhoA/ROCK1 positive cells in the glomerulus was significantly low in the IT group compared to corresponding proportions in the DN group (Figures 4(e)–4(g)). The expression levels of RhoA/ROCK1 proteins were significantly low in the IT group compared to the corresponding levels in the DN group (Figures 4(h) and 4(i)). Evidently, IT treatment reduced the expression of RhoA/ROCK1 but increased the expression of synaptopodin in the glomeruli.

### 3.5. Inhibition of RhoA/ROCK1 Improves Recovery of Podocyte Injury Induced by High Glucose

**Figure 5:**
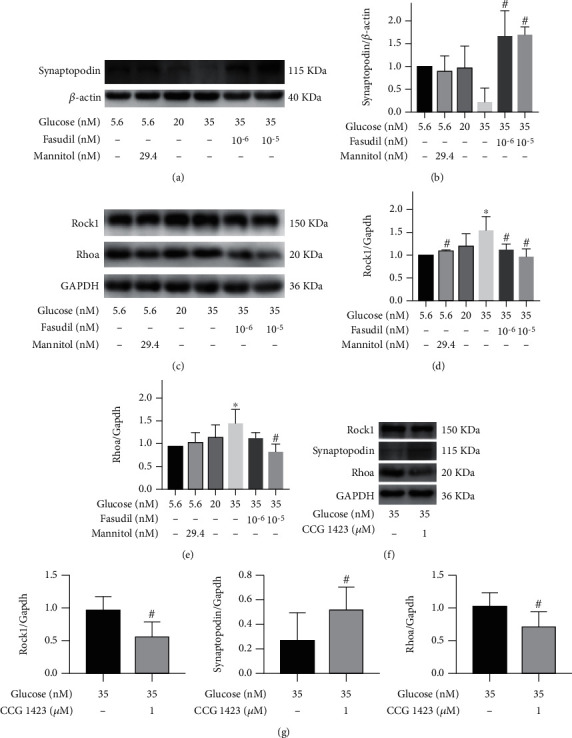
Effects of HG and fasudil on synaptopodin and RhoA/ROCK1 proteins in podocytes in vitro. (a, b) The dose-dependent effects of HG and fasudil on synaptopodin protein and their semiquantitative analysis. (c–e) The dose-dependent effects of HG and fasudil on RhoA/ROCK1 proteins and their semiquantitative analysis. (f, g) The effects of CCG-1423 on synaptopodin and RhoA/ROCK1 proteins in HG (35.0 mmol/L glucose) and heir semiquantitative analysis. ^∗^*P* < 0.05 versus the control group (5.5 mM glucose), ^#^*P* < 0.05 versus the HG (35 mM) alone.

The degree of injury of immortalized podocytes was determined at different glucose concentrations to assess whether inhibition of the RhoA/ROCK1 pathway was related to the degree of podocyte recovery. The expression levels of synaptopodin and RhoA/ROCK1 proteins were subsequently measured by WB. Podocytes was decreased when the glucose concentration was high (i.e., 20.0 and 35.0 mmol/L) and vice versa (Figures 5(a) and 5(b)). In the same line, mannitol had no effect on podocyte expression and activation of the RhoA/ROCK1 signaling pathway. Mannitol had been used as an osmotic control. However, fasudil reduced the increased expression level of RhoA/ROCK1 proteins induced by high glucose (35.0 mmol/L glucose) levels (Figures 5(c)–5(e)). Fasudil also restored the expression of podocytes in high glucose (35.0 mmol/L glucose; Figures 5(a) and 5(b)). Similarly, CCG-1423 also restored the expression of podocytes and reduced the increased expression level of RhoA/ROCK1 proteins in high glucose (35.0 mmol/L glucose; Figures 5(f) and 5(g)).

### 3.6. Islet Cells Reverse Podocyte Injury Caused by High Glucose by Inhibiting the RhoA/ROCK1/NF-*κ*B Pathway

**Figure 6:**
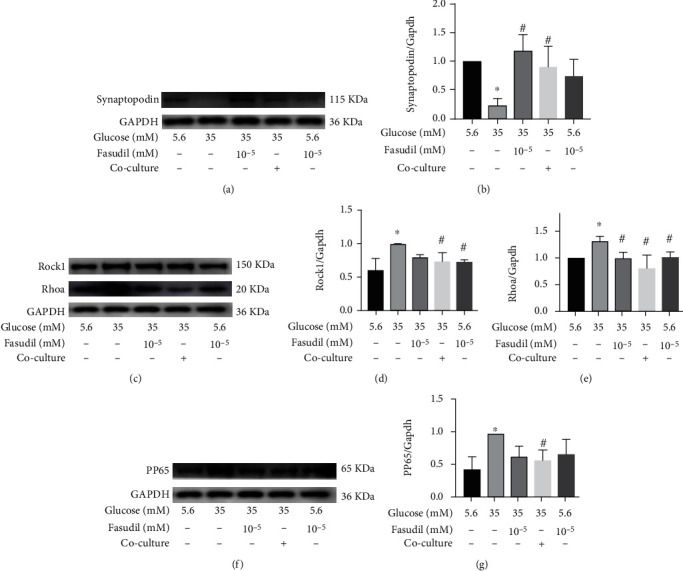
Effects of islet cells and fasudil on synaptopodin, RhoA/ROCK1and pp65 proteins of podocyte in high glucose (35.0 mmol/L glucose) environment. (a, b) The effects of islet cells (islet cells were cocultured with podocytes) on synaptopodin protein and their semiquantitative analysis. (c–e) The effects of islet cells on proteins of RhoA/ROCK1 signaling pathway and their semiquantitative analysis. (f, g) The effects of islet cells and fasudil on pp65 protein and their semiquantitative analysis. ^∗^*P* < 0.05 versus the control group (5.5 mM glucose), ^#^*P* < 0.05 versus the HG (35 mM) alone. Coculture group: islet cells were cocultured with podocytes.

The effects of high glucose (35.0 mmol/L glucose), fasudil, and islet cells (islet cells cocultured with podocytes) on the expression of RhoA/ROCK1, PP65, and synaptopodin were determined by WB *in vitro* to identify the molecular mechanisms of islet transplantation in reversing podocyte injury in diabetic nephropathy. Both islet cells and fasudil attenuated the expression of RhoA/ROCK1 proteins and restored podocyte expression in high glucose (35.0 mmol/L glucose) conditions (Figures 6(a)–6(e)). Moreover, the increased expression of pp65 induced by HG (35.0 mmol/L glucose) was inhibited by fasudil. This indicated that NF-*κ*B expression was mediated by the RhoA/ROCK signaling pathway (Figures 6(f) and 6(g)). These findings demonstrated that islet cells and fasudil reversed the HG (35.0 mmol/L glucose) induced low synaptopodin expression by inhibiting expression of the RhoA/ROCK1/NF-*κ*B pathway.

## 4. Discussion

Herein, the role of islet transplantation in reversing podocyte injury was investigated. The diabetic nephropathy model was established, and islet cells successfully isolated before transplantation. After islet transplantation, the blood glucose of DN mice decreased significantly and remained constant within the normal level. Moreover, the body weight gradually increased, and the symptoms of polyuria disappeared. The renal function index (blood creatinine, urea nitrogen, and urinary albumin-to-creatinine) was also improved, and the damage to the kidney tissue (especially podocyte) significantly alleviated. Further exploration of the mechanism involved revealed that the RhoA/ROCK1 signaling pathway was closely related to podocyte injury in diabetic nephropathy. Nevertheless, the pathway was significantly inhibited after islet transplantation. These results were confirmed using cell experiments. Notably, the therapeutic effect of islet cells and fasudil was related to suppression of pp65 (NF-*κ*B p65 subunit) activity. It was therefore postulated that the RhoA/ROCK/NF-*κ*B pathway is involved in podocyte repair after islet transplantation.

Islet transplantation is a radical treatment for type 1 diabetes mellitus. It can restore insulin secretory capacities and increase insulin sensitivity [13, 14]. Pancreatic islet grafts can ameliorate diabetic angiopathy and prevent its progression to diabetic nephropathy [6]. Previous studies have reported that islet transplantation can improve podocyte injury via Notch-1 signaling. However, other mechanisms of podocyte repair have not yet been deeply studied [7, 15]. In previous islet transplantation studies, there were individual differences in the degree of injury after diabetic nephropathy modeling and also individual differences in repair of kidney injury after islet transplantation. Moreover, an abnormal signal activation was detected in the kidneys of the animals with poor recovery after islet transplantation. As such, focus was put on the effect of the abnormal signal on the repair of podocytes.

Podocytes are highly differentiated postmitotic cells that play a critical role as filtration barriers and prevention of protein loss [16]. They are damaged in the presence of diabetic nephropathy or high glucose conditions [17]. Podocyte abnormalities are also believed to contribute to the development of proteinuric glomerular diseases [18]. In the same line, their injury leads to albuminuria and other typical pathological changes such as glomerular hypertrophy, glomerulosclerosis, and GBM thickening which further aggravate the development of diabetic nephropathy [19]. As such, repair of podocytes is of great significance in DN treatment. Synaptopodin (podocyte-specific marker) is specifically expressed in differentiated podocytes. Its expression level reflects the injury degree of the podocytes and is therefore commonly used in podocyte and diabetic nephropathy research [20].

Filamentous actin rearrangements and damage in podocytes are related to the activation of ROCK. They are usually induced by high glucose [21]. Activated RhoA in turn induces podocyte apoptosis through TGF*β* [22]. During pathogenesis of DN, there is high expression of RhoA/ROCK1 [11]. It is postulated that the RhoA/ROCK signal can upregulate NF-*κ*B activity which plays a key role in the occurrence and development of DN [23–25]. MCP-1 is a member of the C-C chemokines. It is regulated by NF-*κ*B signaling and is highly expressed in many renal diseases (especially in DN) [26]. These reports confirm the role of inflammatory pathways in development of DN [27]. Herein, the highly expressed RhoA/ROCK signal in podocytes of DN was suppressed by IT. In addition, IT significantly increased the expression of synaptopodin. IT also inhibited the expression of inflammatory factors IL-6 and MCP-1 mediated by NF-ĸB in the kidney tissues of DN mice. These findings strongly suggested that IT could reverse podocyte injuries in diabetic nephropathy by inhibiting the RhoA/ROCK/NF-*κ*B pathway.

These findings were further confirmed using *in vivo* studies. Podocyte injuries were significantly reversed, and the expression of the RhoA/ROCK1 signal inhibited in the high-glucose environment after the cells were cocultured with islet cells. Similarly, fasudil (a ROCK inhibitor) [28] alleviated the podocyte injuries induced by high glucose. These results were consistent with those of Hidaka Teruo et al., who also reported that fasudil had beneficial effects on podocyte injury induced by angiotensin II [29]. Moreover, we found that NF-*κ*B was activated in podocyte injury, and they were also consistent with those of Qinglian Wang et al., who reported that NF-*κ*B activated because of podocyte injury was mediated by RhoA/ROCK signalling pathway [30]. CCG-1423 (RhoA Inhibitor) also yielded similar experimental results which further validated the findings. It was thus postulated that islet cells can reverse the podocyte injury induced by high glucose by inhibiting the RhoA/ROCK/NF-ĸB pathway.

Activation of abnormal renal signals will affect the repair of podocyte injury after islet transplantation. However, so far, the treatment in this aspect is very limited. Herein, inhibition of the RhoA/ROCK/NF-ĸB pathway after islet transplantation had a great protective effect on podocyte injury in diabetic nephropathy which further enriched the therapeutic regimen of islet transplantation.

Nonetheless, this study was limited by several factors. The study lacked a fasudil treatment group. As such, the clinical value of islet transplantation combined with fasudil in treatment of diabetic nephropathy needs to be explored further using large comprehensive studies. In addition, NF-*κ*B could have also caused inflammatory podocyte injury. This phenomenon should therefore be investigated in the future studies.

## 5. Conclusion

Evidently, islet transplantation can reverse podocyte injury in diabetes nephropathy by inhibiting the RhoA/ROCK1 signaling pathway. Islet cells have a strong protective effect on podocytes treated with high glucose. Discovery of signaling pathways affecting podocyte recovery is helpful for individualized efficacy evaluation and targeted therapy of islet transplantation patients.

## Data Availability

All data is available from the corresponding author upon request.

## Abbreviations

HG: High glucose
DN: Diabetic nephropathy
IT: Islet transplantation
STZ: Streptozotocin
FDA-PI: Fluorescein diacetate-propidium iodide
HE: Hematoxylin-eosin
DAB: Diaminobenzidine
ROCK: Rho kinase
RhoA: Ras homolog gene family, member A
NF-*κ*B: Nuclear factor kappa B
MCP-1: Monocyte chemoattractant protein-1
IL-6: Interleukin-6
IHC: Immunohistochemistry staining
WB: Western blotting.

## References

[1] Shen Z., Fang Y., Xing T., Wang F. (2017). Diabetic nephropathy: from pathophysiology to treatment. *Journal of Diabetes Research*.

[2] Tojo A., Kinugasa S. (2012). Mechanisms of glomerular albumin filtration and tubular reabsorption. *International Journal of Nephrology*.

[3] Kim J. S., Han B. G., Choi S. O., Cha S. K. (2016). Secondary focal segmental glomerulosclerosis: from podocyte injury to glomerulosclerosis. *BioMed Research International*.

[4] Fioretto P., Steffes M. W., Sutherland D. E. R., Goetz F. C., Mauer M. (1998). Reversal of lesions of diabetic nephropathy after pancreas transplantation. *The New England Journal of Medicine*.

[5] Fiorina P., Folli F., Zerbini G. (2003). Islet transplantation is associated with improvement of renal function among uremic patients with type I diabetes mellitus and kidney transplants. *Journal of the American Society of Nephrology : JASN*.

[6] Fiorina P., Venturini M., Folli F. (2005). Natural history of kidney graft survival, hypertrophy, and vascular function in end-stage renal disease type 1 diabetic kidney-transplanted patients: beneficial impact of pancreas and successful islet cotransplantation. *Diabetes Care*.

[7] He Y., Zhang M., Wu Y. (2018). Aberrant activation of Notch-1 signaling inhibits podocyte restoration after islet transplantation in a rat model of diabetic nephropathy. *Cell Death & Disease*.

[8] Bose M., Almas S., Prabhakar S. (2017). Wnt signaling and podocyte dysfunction in diabetic nephropathy. *Journal of Investigative Medicine : the Official Publication of the American Federation for Clinical Research*.

[9] Etienne-Manneville S., Hall A. (2002). Rho GTPases in cell biology. *Nature*.

[10] Kolavennu V., Zeng L., Peng H., Wang Y., Danesh F. R. (2008). Targeting of RhoA/ROCK signaling ameliorates progression of diabetic nephropathy independent of glucose control. *Diabetes*.

[11] Peng F., Wu D., Gao B. (2008). RhoA/Rho-kinase contribute to the pathogenesis of diabetic renal disease. *Diabetes*.

[12] Lembert N., Wesche J., Petersen P., Doser M., Becker H. D., Ammon H. P. (2003). Areal density measurement is a convenient method for the determination of porcine islet equivalents without counting and sizing individual islets. *Cell Transplantation*.

[13] Hirsch D., Odorico J., Radke N. (2010). Correction of insulin sensitivity and glucose disposal after pancreatic islet transplantation: preliminary results. *Diabetes, Obesity & Metabolism*.

[14] Zhu X., Guo F., Tang H. (2019). Islet transplantation attenuating testicular injury in type 1 diabetic rats is associated with suppression of oxidative stress and inflammation via Nrf-2/HO-1 and NF-*κ*B pathways. *Journal of Diabetes Research*.

[15] He Y., Xu Z., Zhou M. (2016). Reversal of early diabetic nephropathy by islet transplantation under the kidney capsule in a rat model. *Journal of Diabetes Research*.

[16] Kriz W., Hackenthal E., Nobiling R., Sakai T., Elger M., Hähnel B. (1994). A role for podocytes to counteract capillary wall distension. *Kidney International*.

[17] Fiorina P., Vergani A., Bassi R. (2014). Role of podocyte B7-1 in diabetic nephropathy. *Journal of the American Society of Nephrology : JASN*.

[18] Barisoni L., Schnaper H. W., Kopp J. B. (2007). A proposed taxonomy for the podocytopathies: a reassessment of the primary nephrotic diseases. *Clinical Journal of the American Society of Nephrology : CJASN*.

[19] Li J. J., Kwak S. J., Jung D. S. (2007). Podocyte biology in diabetic nephropathy. *Kidney International Supplement*.

[20] Mundel P., Reiser J., Borja A. Z. Ḿ. (1997). Rearrangements of the cytoskeleton and cell contacts induce process formation during differentiation of conditionally immortalized mouse podocyte cell lines. *Experimental Cell Research*.

[21] Lv Z., Hu M., Ren X. (2016). Fyn mediates high glucose-induced actin cytoskeleton reorganization of podocytes via promoting ROCK activation in vitro. *Journal of Diabetes Research.*.

[22] Babelova A., Jansen F., Sander K. (2013). Activation of Rac-1 and RhoA contributes to podocyte injury in chronic kidney disease. *PLoS One*.

[23] Shimada H., Rajagopalan L. E. (2010). Rho kinase-2 activation in human endothelial cells drives lysophosphatidic acid-mediated expression of cell adhesion molecules via NF-kappaB p65. *The Journal of Biological Chemistry*.

[24] Xie X., Chang X., Chen L. (2013). Berberine ameliorates experimental diabetes-induced renal inflammation and fibronectin by inhibiting the activation of RhoA/ROCK signaling. *Molecular and Cellular Endocrinology*.

[25] Xie X., Peng J., Chang X. (2013). Activation of RhoA/ROCK regulates NF-*κ*B signaling pathway in experimental diabetic nephropathy. *Molecular and Cellular Endocrinology*.

[26] Wang J. C., Zhao Y., Chen S. J. (2013). AOPPs induce MCP-1 expression by increasing ROS-mediated activation of the NF-*κ*B pathway in rat mesangial cells: inhibition by sesquiterpene lactones. *Cellular Physiology and Biochemistry : International Journal of Experimental Cellular Physiology, Biochemistry, and Pharmacology*.

[27] Mora C., Navarro J. F. (2004). Inflammation and pathogenesis of diabetic nephropathy. *Metabolism: Clinical and Experimental*.

[28] Olson M. F. (2008). Applications for ROCK kinase inhibition. *Current Opinion in Cell Biology*.

[29] Hidaka T., Suzuki Y., Yamashita M. (2008). Amelioration of crescentic glomerulonephritis by RhoA kinase inhibitor, Fasudil, through podocyte protection and prevention of leukocyte migration. *The American Journal of Pathology*.

[30] Wang Q., Shen Z., Qi G., Zhao Y., Zhang H., Wang R. (2020). Thymol alleviates AGEs-induced podocyte injury by a pleiotropic effect via NF-*κ*B-mediated by RhoA/ROCK signalling pathway. *Cell Adhesion & Migration*.

